# 3D analysis of motion in the non-united elbow and deformity in the malunited forearm using a computerized simulation system

**DOI:** 10.1186/1753-6561-9-S3-A93

**Published:** 2015-05-19

**Authors:** Eugene Kim

**Affiliations:** 1 Department of Orthopaedic Surgery, University School of Medicine, Seoul, 136-705, South Korea

## Introduction

Three-dimensional image data of the human body can provide much information to the medical field and 3D simulated navigation surgery is popular in total knee arthroplasty. However, very little study has been conducted in the upper extremity including the hand.

Non union of the lateral condyle fracture combined with cubitus valgus deformity often results in a painful elbow together with a cosmetic problem. However the treatment is still controversial. Corrective osteotomy for valgus deformity is not physiologic and a high rate of complication has also been reported in osteosynthesis. We attempt to analyze the motion of the elbow and pseudo-joint of a non-united lateral condyle.

Traumatic plastic bowing deformity of the forearm in a Monteggia fracture dislocation has focused on corrective bowing in plane radiographs 2 dimensionally or reduction only. Few 3-dimensional biomechanical investigations of plastic bowing deformity has been reported and we intend to analyze deformity 3-dimensionally.

The authors introduce a 3D motion analysis and 3D deformity analysis using computer simulation with the goal of this study to elucidate appropriate treatments with biomechanical consideration.

## 3D image processing and analyze

6 non united lateral condyle of elbows and 10 traumatic plastic deformity of the ulna with chronic radial head dislocations (Monteggia equivalent) were performed with CT scans. Non united lateral condyle cases were scanned at three different degrees of elbow position (full flexion, 90° flexion and full extension). Plastic deformity cases were scanned on both side of forearms.

**Figure 1:**
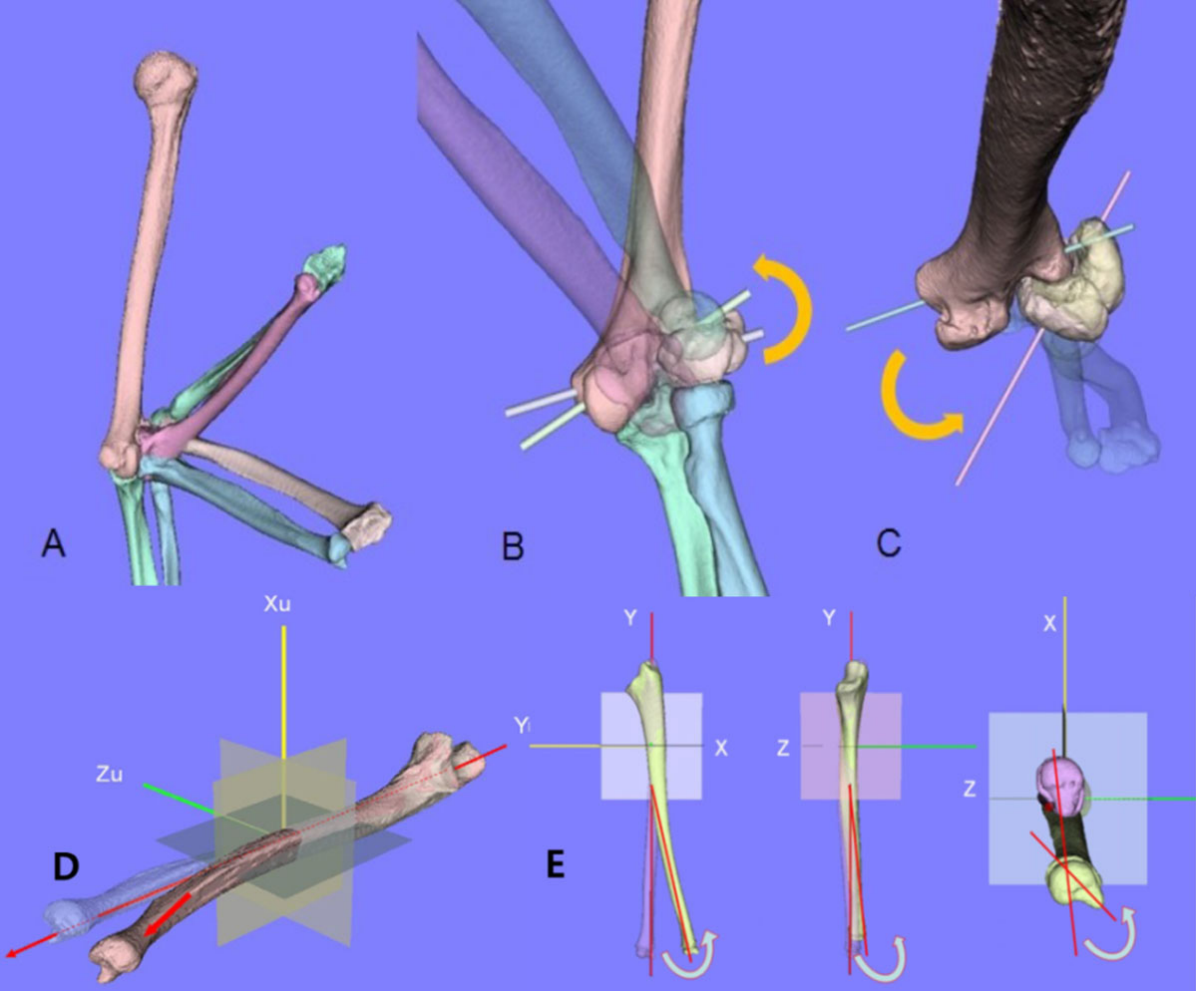
3D motion of non united elbow and 3D deformity of plastic bowing deformity of forearm

First, we used a simulation program (Bone Viewer™, Orthree Co. Ltd., Osaka, Japan) to construct three-dimensional surface models of the humerus, ulna, radius and non united fragment. (Fig 1)

Second, in the non-united elbow, we superimposed a total of three position of the humerus based on semiautomatic markerless surface-based registration technique using a software system (Bone Simulator™, Orthree Co. Ltd., Osaka, Japan). We then calculated the rotational axis of the humero-ulna joint in the position of full extension to full flexion. Based on the calculation, we analyzed the motion of rotational axis of humero-ulna joint of elbow and pseudojoint of affected humerus and lateral condylar fragment.

In plastic bowing, the proximal part of the affected ulna was superimposed onto its mirror image of the contralateral normal bone.

Then, the amount of 3-dimensional displacement of the distal part of the affected side relative to the mirror image of the normal side was quantified in Euler angles. When the rotational angles of the distal parts of the ulna and radius matched their mirror images, this was considered to indicate the deformity angle in 3 directions. Bending deformity in the sagittal or coronal plane together with rotational deformity in the axial plane was quantified.

## Result of3D kinematics and 3D deformity

In the non-united elbow, the rotational axis of the normal elbow showed minimal changes during flexion like the hinge joint. In the elbow joint of the non-united lateral condyle of the humerus, there was rotation to valgus in the coronal plane and rotation externally in the axial plane. Movement of the rotational axis of the bony fragment varies from case to case. In three cases, the lateral condyle moved along a similar course as the elbow. In two cases, it rotated externally in the axial plane, and in one case it moved in the opposite direction (extension during elbow flexion).

Plastic bowing of the ulna with a Monteggia lesion had a 18.7 ±17.4 of external rotation in the axialplane and 10.4 ±7.0 of extension in the sagittal plane. Hence, rotational deformities were larger than those of sagittal and coronal bending deformities.

## Discussion

In the simulation study, the rotational axis of normal elbows demonstrates hinge joint movement without motional change. In cases of non-union, the rotational axis moves during flexion thus resulting in valgus instability and potential destruction of the joint surface. From the simulation results, we confirmed a positive consideration of osteosynthesis of the non-united bony fragment based on the motion analysis of the bony fragment, not only for corrective osteotomy for correction of the valgus deformity but also for reconstruction of stable elbows.

With the 3D computerized simulation system, the surgeon can predict not only gross bending deformity but rotational deformity, therefore the correction of rotational deformities of the ulna should be considered in the treatment of chronic radial head dislocations with deformity. Also, 3D assessment of plastic bowing deformity provides important information on torque and the direction of force of the initial injuries which could yield presumptions regarding the mechanism of the injury. Thus, in injuries due to falls, external rotational stress on the ulna is suspected to cause rotational deformity with radial head dislocation.

In conclusion, 3D computerized simulation system provides important information about 3D deformity and 3D kinematics of joint motion allowing us to pursue better treatment modalities.
